# Towards Arginase Inhibition: Hybrid SAR Protocol for Property Mapping of Chlorinated *N*-arylcinnamamides

**DOI:** 10.3390/ijms24043611

**Published:** 2023-02-10

**Authors:** Andrzej Bak, Jiri Kos, Gilles Degotte, Aleksandra Swietlicka, Tomas Strharsky, Dominika Pindjakova, Tomas Gonec, Adam Smolinski, Pierre Francotte, Michel Frederich, Violetta Kozik, Josef Jampilek

**Affiliations:** 1 Institute of Chemistry, University of Silesia, Szkolna 9, 40-007 Katowice, Poland; 2 Department of Biochemistry, Faculty of Medicine, Masaryk University, Kamenice 5, 625 00 Brno, Czech Republic; 3 Department of Analytical Chemistry, Faculty of Natural Sciences, Comenius University, Ilkovicova 6, 842 15 Bratislava, Slovakia; 4 I2BM, Department of Molecular Chemistry, University Grenoble-Alpes, Rue de la Chimie 570, 38610 Gieres, France; 5 Department of Chemical Drugs, Faculty of Pharmacy, Masaryk University, Palackeho 1946/1, 612 00 Brno, Czech Republic; 6 GiG Research Institute, Pl. Gwarkow 1, 40-166 Katowice, Poland; 7 Laboratory of Medicinal Chemistry, CIRM, Department of Pharmacy, University of Liege, Avenue Hippocrate 15, 4000 Liege, Belgium; 8 Laboratory of Pharmacognosy, CIRM, Department of Pharmacy, University of Liege, Avenue Hippocrate 15, 4000 Liege, Belgium; 9 Institute of Neuroimmunology, Slovak Academy of Sciences, Dubravska cesta 9, 845 10 Bratislava, Slovakia

**Keywords:** arginase inhibition, arylcinnamamides, lipophilicity, CoMSA, molecular docking, similarity-activity landscape index

## Abstract

A series of seventeen 4-chlorocinnamanilides and seventeen 3,4-dichlorocinnamanilides were characterized for their antiplasmodial activity. In vitro screening on a chloroquine-sensitive strain of *Plasmodium falciparum* 3D7/MRA-102 highlighted that 23 compounds possessed IC_50_ < 30 µM. Typically, 3,4-dichlorocinnamanilides showed a broader range of activity compared to 4-chlorocinnamanilides. (2*E*)-*N*-[3,5-bis(trifluoromethyl)phenyl]-3-(3,4-dichlorophenyl)prop-2-en-amide with IC_50_ = 1.6 µM was the most effective agent, while the other eight most active derivatives showed IC_50_ in the range from 1.8 to 4.6 µM. A good correlation between the experimental log*k* and the estimated clogP was recorded for the whole ensemble of the lipophilicity generators. Moreover, the SAR-mediated similarity assessment of the novel (di)chlorinated *N*-arylcinnamamides was conducted using the collaborative (hybrid) ligand-based and structure-related protocols. In consequence, an ‘averaged’ selection-driven interaction pattern was produced based in namely ‘pseudo–consensus’ 3D pharmacophore mapping. The molecular docking approach was engaged for the most potent antiplasmodial agents in order to gain an insight into the arginase-inhibitor binding mode. The docking study revealed that (di)chlorinated aromatic (C-phenyl) rings are oriented towards the binuclear manganese cluster in the energetically favorable poses of the chloroquine and the most potent arginase inhibitors. Additionally, the water-mediated hydrogen bonds were formed via carbonyl function present in the new *N*-arylcinnamamides and the fluorine substituent (alone or in trifluoromethyl group) of *N*-phenyl ring seems to play a key role in forming the halogen bonds.

## 1. Introduction

Due to its facile transmission via the bite of an infected *Anopheles* mosquito, malaria poses a serious threat to health of human beings, especially in the developing countries [1,2]. It still remains one of the most prevalent infectious diseases worldwide caused by protozoan parasites of five *Plasmodium* species that inject the host body in the form of sporozoites. According to statistics, *Plasmodium falciparum* is the most lethal one [3]. At the beginning of infection, the rapid proliferation (extensive replication) of parasite cells is observed in the pathogenic course of the disease, while the host’s hepatocytes in the liver are invaded, merozoites are formed and released into the host’s bloodstream causing the range of malaria symptoms (e.g., hypoglycemia, hemoglobinuria, hemolytic anemia, lactic acidosis) [4,5]. Moreover, clinically malaria is frequently manifested with hypoargininemia—nearly complete depletion of the host l-arginine (Arg) [6]. The decreased level of the host l-Arg is the result of the increased exogenous arginase activity from the malarial parasite, that catalyzes the hydrolysis of the guanidinium Arg side-chain to form urea and l-ornithine, respectively. In fact, Arg can be metabolized using multiple pathways (e.g., nitric oxide synthase NOS), but arginase route is crucial in the production of the sufficient amount of the polyamine synthesis precursor, l-ornithine. In other words, keeping the right l-ornithine level to produce polyamines is essential for *Plasmodium* development and maturation to escalate both the parasite infectivity as well as to reduce the host’s defensive capacity [7,8,9].

Over the last few decades, the manganese-containing arginases (e.g., human hAI and hAII, *Leishmania amazonesis* LA, or *P. falciparum* PFA metalloenzymes) have been established as attractive drug target candidates, that are involved in many human pathophysiological disorders, such as pulmonary hypertension, asthma or cancer [10,11]. In consequence, the range of structurally diverse arginase inhibitors have been tested and implemented clinically so far as therapeutic agents, forming the first generation (e.g., *N^ω^*-hydroxy-l-arginine NOHA or *N^ω^*-hydroxy-nor-l-arginine nor-NOHA) and the second generation (e.g., *S*-(2-boronoetyl)-l-cysteine BEC or 2-(*S*)-amino-6-boronohexanoic acid ABH) of the marketed drugs, respectively [12,13]. Unfortunately, the therapeutic application of the synthetic arginase inhibitors is limited due to the poor bioavailability, potential toxicity, and relatively short half-life of such molecules [14]. Hence, extensive efforts have been undertaken to design new pharmacologically active agents of great therapeutic relevance. One source of inspiration comes from nature, where plants provide natural products (NPs) that can be used as potential (sub)components (lead structures) of novel arginase inhibitors.

A variety of phenolic acid derivatives (e.g., cinnamic acid) and the related natural dihydroxycinnamic compounds (e.g., catechol-containing caffeic, chlorogenic, or rosmarinic acids) revealed the antileishmanial and/or antiplasmodial potency [15,16]. Hence, rosmarinic acid analogues and new caffeic acid-derivative amides were synthesized and tested to target human and *L. amazonesis* arginases, respectively [17,18]. In an attempt to determine the potential arginase-inhibitor binding modes, the computer-aided structure-based protocol was applied indicating that cinnamoyl or 3,4-hydroxycinnamoyl motifs are crucial for the mechanism of arginase inhibition. In consequence, cinnamic acid and its derivatives are promising starting point on the path from molecules to drugs due to their low toxicity and wide spectrum of anti-infective potential [19,20]. In the search for new pharmacologically active agents a number of cinnamic acid anilides were prepared and tested to specify their antifungal, antibacterial, and antimycobacterial activity profile [21,22,23,24,25]. Based on the antileishmanial activity of dihydroxycinnamic compounds, where the catechol-like moiety is frequently involved in a metal (Mn^2+^) coordination bond by one of its hydroxyl groups, we proposed the introduction of halogen into the molecular structure of *N*-arylcinnamammides. As a result of the subsequent chlorination of the benzene ring, two series of anilides based on 4-chlorocinnamic and 3,4-dichlorocinnamic acids were designed, prepared and characterized. Moreover, the empirical lipophilicity of new compounds was determined using high-performance liquid chromatography (HPLC). Typically, 3,4-dichlorocinnamanilides showed a broader range of activity compared to their mono-substituted counterparts (4-chlorocinnamanilides), but all molecules are at least as effective as commercially used drugs (e.g., ampicillin, isoniazid, rifampicin) [26].

According to our best knowledge, the exact mechanism of action of cinnamic acid scaffolds on *P. falciparum* remains unknown. Like the human arginases, *P. falciparum* arginase (PFA) is a binuclear manganese metalloenzyme that exists as a trimer with optimal activity at basic environment [27]. PFA largely shares aminoacid residue (R) sequence with human arginases hAI and hAII; however, it differs considerably from hAI and hAII in so-called low complexity region (LCR) of the loop L2 [28]. Moreover, the structural comparisons of mammalian, bacterial, and parasitic arginases indicated noticeable variations in the stabilization of oligomeric structure [29]. On the other hand, PFA exhibits catalytic efficiency comparable to hAI that is manifested by the similar binding mode of the liganded hAI-ABH complex. In order to gain an insight into the possible mechanism of the parasitic arginase inhibition, the holo-form of PFA-ABH crystal structure might serve as an attractive target to potential antimalarial agents, especially against the liver-stage infection [30].

Luckily, the medicinal chemist’s intuition (or serendipity) can be supported at the decision-making cascade of the hit identification→lead optimization→drug nomination by computer-assisted molecular design (CAMD) in order to predict ADMET-friendly molecular properties and to reduce the probability of a drug’s late attrition according to the ‘fail early fail cheaply’ concept [31]. Hence, a range of in silico methods has been introduced for mapping the molecular topology/topography (encoded with the symbolic/numeric descriptors) into the ADMET-tailored chemical space (CS); however, the straightforward transition from intricate biological relations into simple quantitative structure-activity relationships (QSARs) can ‘lead down a blind alley’ [32]. Despite some shortcomings, SAR-guided mining of descriptor-based space became a ‘rule of thumb’ on the path from data to drugs, especially for structurally alike molecules. Unarguably, the core of many SAR-related approaches is molecular similarity—the idea of specifying a numerical measure (metric) of the inter-molecular similarity [33]. Basically, the computer-aided manipulation of the drug-receptor interactions can be dichotomized into ‘indirect’ (ligand-based) and ‘direct’ (structure-based) procedures [34]. Unfortunately, there is no a priori guideline for searching promising drug molecules; therefore, the hybrid (integrated) approach is advisable [35]. Theoretically, the receptor-independent (RI) approach stems loosely from the similarity principle, where steric/electronic/lipophilic-alike interchangeable substituents are bound to exert a similar impact on the pharmacological profile (neighbor behaviors) [36]. In practice, the ‘reverse image’ of the hypothetical target binding mode is produced in the form of spatial (3D) pharmacophoric pattern for the set of structurally related (bio)molecules [37]. In medicinal/computational chemistry, a range of 3D-QSAR procedures have been implemented practically that engages the molecular interaction/energy field (e.g., CoMFA) or molecular surface/volume (e.g., CoMSA) descriptors, respectively. CoMSA replaces the steric (Lennard–Jones) and electrostatic (Coulomb) potential values calculated at single points of CoMFA mesh by the mean potential values specified for surface sectors using self-organizing maps (SOM)—the ‘fuzzification’ of the molecular shape representation is achieved that might provide more realistic picture of the ligand-target recognition scenario [38,39].

In QSAR studies, the optimal balance between ADMET-driven properties and expected drug potency profile can be rationalized graphically by extension of the planar (2D) similarity-driven projection with the activity data in the form of the ‘response surface’ [40]. Detection of a ‘fragile event’ (called ‘activity cliffs’), when even a tiny structural modification (termed a ‘magic methyl’) can boost or completely demolish the biological activity depends critically on the availability of the structurally alike molecules (chemotypes) with discernible activity variations [41]. The systematic profiling (numerical quantification) of the structure-activity landscape indexes (SALI) delivers a subtle picture of (un)favorable structural modifications in order to modulate pharmacological response of the potent drug candidates [42,43]. The distance-oriented property evaluation can be performed using the linear (e.g., principal component analysis PCA, hierarchical clustering analysis HCA) or/and non-linear (e.g., self-organizing maps SOMs) data reduction (DR) procedures in order to investigate the (dis)similarities between objects (molecules) in the multidimensional descriptor-based space [44].

The qualitative and/or quantitative rationalization of the drug-target binding forces in the receptor-dependent (RD) approach can be partially deduced using the site-directed molecular docking approach, especially beneficial when a spatial geometry (or homology model) of the target binding site is accessible [45,46]. In fact, the utility of the intuitive docking procedures for producing the guest-host poses (ligand conformations and orientations) in the structure-based drug design is widely accepted as a complimentary protocol to the classical ligand-driven methods, respectively.

In the current paper, the collaborative (hybrid) protocol for property mapping of novel (di)chlorinated *N*-arylcinnamamides as potential PFA inhibitors is reported; therefore, in vitro screening of the library of 35 new compounds on a chloroquine-sensitive strain of *P. falciparum* 3D7/MRA-102 was performed. Then, a SAR-mediated similarity assessment of the structural descriptors and experimental data (inhibitory potential and lipophilic profile) for the new molecules was conducted using PCA and HCA methods. In order to predict the activity cliffs SALI indexes were calculated as well. Moreover, the quantitative atom-based (CoMFA) and shape-related (CoMSA) ligand-oriented sampling of inter-molecular similarity and enzyme-driven molecular docking of ligands into the target pocket (active site) were applied to specify the electronic/steric/lipophilic factors and the ligand–enzyme (bio)composition that are potentially valid for the structure-inhibitory potency modeling of new PFA inhibitors. The stochastic model validation (SMV) was used to generate the probabilistic CoMSA pharmacophore pattern.

Furthermore, the molecular docking approach was engaged for the most potent antiplasmodial agents in order to obtain the comprehensive knowledge of the arginase-inhibitor binding mode. The docking study revealed that (di)chlorinated aromatic (C-phenyl) rings are oriented towards the binuclear manganese cluster in the energetically favorable poses of the chloroquine (CQ) and the most potent arginase inhibitors. Additionally, the water-mediated hydrogen bonds were formed via carbonyl function present in the new *N*-arylcinnamamides. Interestingly, the fluorine substituent (alone or in trifluoromethyl group) of *N*-phenyl seems to play a key role in forming the halogen bonds between arginase and the most potent inhibitors. As a matter of fact, the collaborative combination of the pharmacophore mapping with target-tailored protocols can help to modulate pharmacological response and optimize ADMET-friendly drug properties to produce potentially more potent antiplasmodial drug candidates.

## 2. Results

### 2.1. Lipophilic and Antiplasmodial Activity Profiles Evaluation

**Scheme 1:**
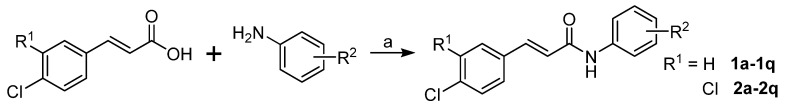
Synthesis of (*2E*)-3-(4-chlorophenyl)-*N*-arylprop-2-enanilides (**1a**-**1q**) and (*2E*)-3-(3,4-dichlorophenyl)-*N*-arylprop-2-enanilides (**2a**-**2q**). *Reagents and conditions*: (**a**) PCl*_3_*, chlorobenzene, MW, 40 min [26].

Sets of seventeen ring-substituted (2*E*)-3-(4-chlorophenyl)-*N*-arylprop-2-enanilides (series **1a**–**1q**) and seventeen (2*E*)-3-(3,4-dichlorophenyl)-*N*-arylprop-2-enanilides (series **2a**–**2q**) were synthesized (Scheme 1) and characterized in our previous study [26].

In the current study, all newly synthesized 4-chlorocinnamanilides and 3,4-dichlorocinnamanilides were evaluated for their in vitro antiplasmodial activity. Moreover, the lipophilic profile of the compounds was experimentally determined using the RP-HPLC technique [26], as shown in Table 1. Typically, 3,4-dichlorocinnamanilides showed a broader range of activity compared to 4-chlorocinnamanilides.

### 2.2. Biological Potency Profiling

#### 2.2.1. Antiplasmodial Activity Evaluation

In vitro screening of the library of thirty-four compounds on a CQ-sensitive strain of *P. falciparum* 3D7/MRA-102 highlighted, that 23 compounds possessed an IC_50_ < 30 µM and could be considered as active antiplasmodial agents (see Table 1). The most effective compounds were the following: (2*E*)-*N*-[3,5-bis(trifluoromethyl)phenyl]-3-(3,4-dichloro- phenyl)prop-2-enamide (**2p**, IC_50_ = 1.6 µM), (2*E*)-3-(3,4-dichlorophenyl)-*N*-(3,5-difluoro- phenyl)prop-2-enamide (**2l**, IC_50_ = 1.8 µM), (2*E*)-3-(3,4-dichlorophenyl)-*N*-(2,4-difluoro- phenyl)prop-2-enamide (**2k**, IC_50_ = 1.9 µM) and (2*E*)-*N*-[3,5-bis(trifluoromethyl)phenyl]- 3-(4-chlorophenyl)prop-2-enamide (**1p**, IC_50_ = 2.5 µM). Other compounds **2m**, **2c**, **2o**, **1o** and **1m** showed IC_50_ in the range from 3.4 to 4.6 µM.

As can be seen, compounds substituted mainly with lipophilic and electron-withdrawing substituents were active. A correlation between antiplasmodial activity and lipophilicity has already been reported for 3,4-dihydroxycinnamic (caffeic) acid [47]. This suggested that the compounds possessing a higher lipophilicity value could more easily enter inside the erythrocytes, reaching higher intracellular concentrations to exert their pharmacological effects [48]. It was also demonstrated that this potency increment seemed to be limited to an optimum lipophilicity value after which the activity stagnates or even decreases because of a higher affinity of the product for the cell membrane compared to the cytosol [49]. In particular, the dependence of the potency to the lipophilicity of the molecules until an optimum log*k* value, suggesting an increased permeation rate, was already reported for other cinnamic acid derivatives [50]. It is important to mention that similar dependences on lipophilicity were also obtained for anilides unsubstituted on the cinnamic core [51]. In addition, the substitution pattern of the *N-*aryl seemed crucial for the anti-*Plasmodium* effect since the most efficient structures possessed at least two different halogen substituents. This suggests that the impact of the substitution on the electronic density of the cycle, as well as on the lipophilicity, is significant on the antiplasmodial effect.

#### 2.2.2. In Vitro Cytotoxicity and Hemolytic Potential

Human monocytic leukemia cells THP-1 were used for determination of the influence of the test derivatives on viability of eukaryotic cells. In vitro cytotoxicity was expressed as IC_50_ values (see Table 1). The results showed that no cytotoxic effects were observed up to a compound concentration of 10 µM [26]. It follows that all the highly anti-*Plasmodium* effective compounds demonstrated insignificant cytotoxicity.

The (di)chlorocinnamanilides were evaluated for their hemolytic potential to confirm the observed in vitro anti*-Plasmodium* potency. Indeed, compounds inducing erythrocyte membrane disruption will cause a significant decrease in the parasitic growth because of its intracellular development. As a result, none of the tested products exhibited any hemolytic activities, confirming their antiplasmodial potential.

### 2.3. In Silico Property Mapping and SAR Screening

#### 2.3.1. Lipophilic Profile Assessment

**Figure 1:**
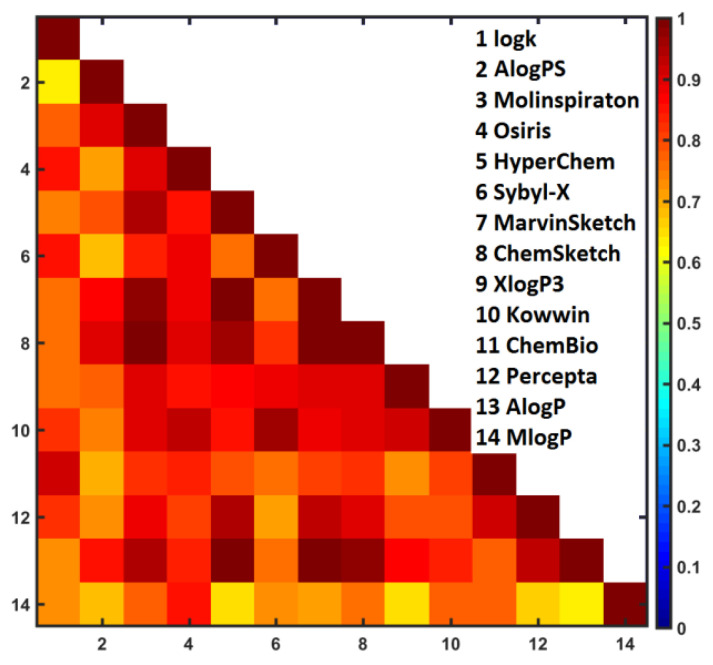
Matrix of correlation coefficients of linear relationships between experimental lipophilicity (log*k*) and calculated lipophilicity (clogP) using different programs.

Molecular lipophilicity is one of the most valid physicochemical property that affects not only the first step of drug action (pharmaceutic phase), but also the drug transport (pharmacokinetics) as well as the host–target binding interactions (pharmacodynamics) [52]. Moreover, early lipophilicity profiling (theoretical and/or empirical) might facilitate better decision-making at early stages of drug design/development so as to eliminate bad actors (false positive hits). Thus, a range of meaningful in silico lipophilicity-based pre-filters was proposed to limit the values of structural or physicochemical descriptors to ADMET-friendly property space, e.g., Lipinski’s Rule of Five (Ro5) for orally administered drugs [53]. Consequently, the lipophilicity of the studied compounds was determined empirically using HPLC technique, as shown in Table 1. In order to profoundly investigate the lipophilic characteristics of new derivatives, the additional in silico approximation of numerical clogP values was conducted using a range of software-based clogP predictors including AlogPS, Molinspirations, Osiris, HyperChem 7.0, Sybyl-X, MarvinSketch 15, ACD/ChemSketch 2015, Dragon6.0, Kowwin, XlogP3, ChemBioDraw, ACD/Percepta. Moreover, the theoretically estimated partition coefficients (clogP) were (inter-)correlated with each other and cross-compared with the experimentally HPLC-specified lipophilic log*k* parameters, as presented in Figure 1.

High correlation between the estimated clogP and experimental log*k* (ranging from r = 0.63 to r = 0.91 with r_mean_ = 0.85 and r_median_ = 0.84) was recorded for the whole ensemble of clogP generators with r > 0.85 calculated for ChemSketch, Sybyl-X HyperChem and Percepta programs, respectively. Despite some variations in clogP values, that are largely dependent on different computational algorithms (e.g., descriptor-based, atom/fragment-related) implemented in the software and/or the modeling data applied at the training stage, the satisfactory inter-correlations between clogP estimators (r ≈ 0.90) were recorded (see Figure 1). In order to indicate the valid set of clogP generators for the new set of molecules the PLS-based methodology with the iterative variable elimination (IVE) was employed on the experimental log*k* data and the integrated clogP matrix (X_34×13_) [54]. The backward elimination with the IVE-PLS procedure indicated that HyperChem, Sybyl-X, ChemSketch, ChemBioDraw and Kowwin property predictors contribute significantly to the final lipophilic model (q^2^_CV_ = 0.89, q^2^_test_ = 0.93). Moreover, the mean values of the selected molecular descriptors that average over the chosen calculation methods were subsequently correlated with the experimental log*k* parameter, namely *consensus* clogP, with a correlation coefficient of 0.88. It should be emphasized, that the balanced selection of clogP estimators prevents the overfitting phenomenon by covering the vast spectrum of theoretical procedures—not only the best (inter) correlated.

#### 2.3.2. Similarity-Based Property Evaluation

**Figure 2:**
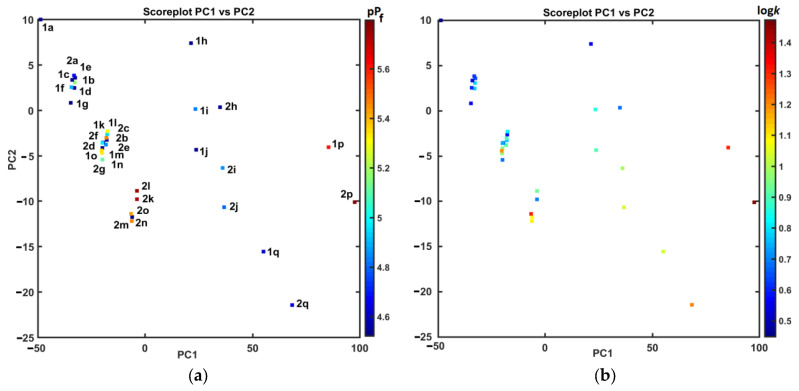
Projection of 4-chlorocinnamanilides **1a**-**q** and 3,4-dichlorocinnamanilides **2a**-**q** on plane defined by PC1 and PC2 color-coded with antiplasmodial activity in logarithmic scale (**a**) and experimental lipophilicity (**b**).

The clustering tendency of the structural descriptor-based data can be traced by analyses of the (dis)similarities between objects/molecules in the multidimensional (mD) variable space; therefore, the distance-related property mapping was performed using the Principal Component Analysis (PCA) and Hierarchical Clustering Analysis (HCA) on the pool of 2804 descriptors generated by Dragon 6.0 software. The obtained data were organized into matrix X_34×2804_ with rows representing objects (molecular series **la**–**q** and **2a**–**q**) and columns representing in silico descriptors (parameters). The resulting matrix was centered and standardized, because the numerical parameters differ considerably. The percentage of the modeled data variance was taken into consideration to calculate the relevant number of the principal components (PCs). The first three PCs describe almost 84% of the total data variance, while the first two PCs account of 68%. The projections (scoreplot) of molecules **1a**–**q** and **2a**–**q** on the plane defined by PC1 vs. PC2, additionally color-coded according to the antiplasmodial activity and the empirical lipophilicity (log*k*) are presented in Figure 2.

Bafflingly, the most active di-substituted with trifluoromethyl (3,5-CF_3_) moieties of *N*-phenyl ring in molecules **1p** and **2p** are located separately (PC1 > 75) from the rest of the derivatives and are characterized by relatively high lipophilic values (log*k* > 1.3), as indicated in Figure 2a,b, respectively. Noticeably, the inactive 4-chlorocinnamanilides **1**(**i**,**j**,**h**,**q**) and their 3,4-dichlorinated in C-phenyl ring counterparts **2**(**i**,**j**,**h**,**q**) are placed in the range of 0 < PC1 < 75. The interesting distribution is observed for the remaining molecules clustered in three sub-groups, where the antiplasmodial activity diminishes with the decrease in PC1 and the parallel increase in PC2, heading to the unsubstituted (R^2^ = H) compounds **1a** and **2a**.

**Figure 3:**
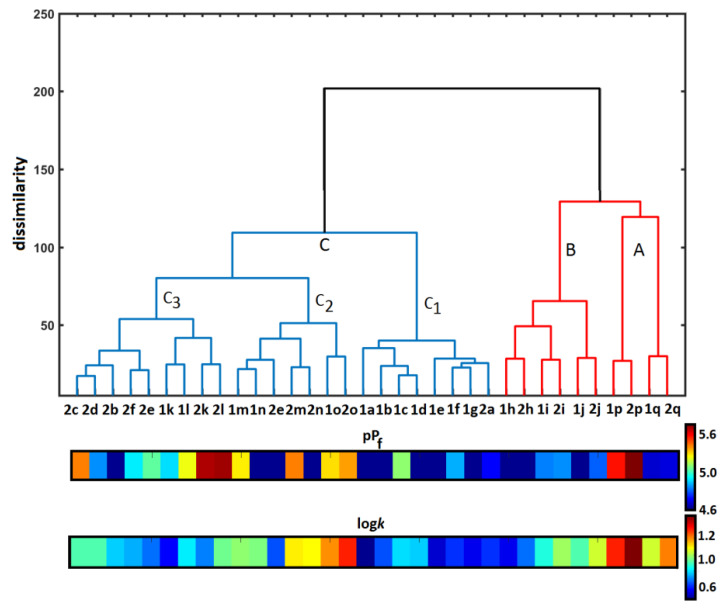
Dendogram of molecules **1a**-**q** and **2a**-**q** in descriptor-based space with color-coded map of biological and lipophilic data in logarithmic scale. Clusters are marked by capital letters.

In order to investigate the (dis)similarity between objects (molecules) in the multidimensional descriptor-based space and the related molecular property profile (e.g., biological or lipophilic characteristics), the findings of Hierarchical Clustering Analysis (HCA) were combined with a color-coded vector of the experimental data, as shown in Figure 3. Briefly speaking, the exploratory HCA procedure generates the sub-optimal clustering pattern of objects that is mainly dependent on the clusters’ linkage procedure employed. In fact, the similarity distance metrics (e.g., Euclidean measure) and the manner of the resulting sub-clusters linkage (e.g., Ward’s algorithm) are selected a priori. In practice, the integration of HCA findings with the colorful display of empirical datasets (e.g., biological activities and lipophilic characteristics) enables the direct interpretation of the produced clusters in the reduced 2D space of the original multidimensional parameters, where OX illustrates the order of objects and OY axis presents the dissimilarity, respectively.

On the whole, the exploratory HCA approach produced the clustering pattern, where molecules are clustered into three main groups (A, B and C), that confirms our previous PCA findings (see Figure 2a). Likewise, the most active 3,5-CF_3_ containing molecules **1p** and **2p** are grouped together in cluster A and are marked by higher values of lipophilicity. Noticeably, the inactive 4-chlorocinnamanilides **1**(**i**,**j**,**h**) and their 3,4-dichlorinated counterparts **2**(**i**,**j**,**h**) are placed in clusters B and C_1_, that are characterized by lower values of lipophilicity. Similar to PCA findings, the remaining molecules are clustered in two sub-groups (C_2_ and C_3_), that are generally described by higher values of the antiplasmodial activity and molecular lipophilicity as well.

**Figure 4:**
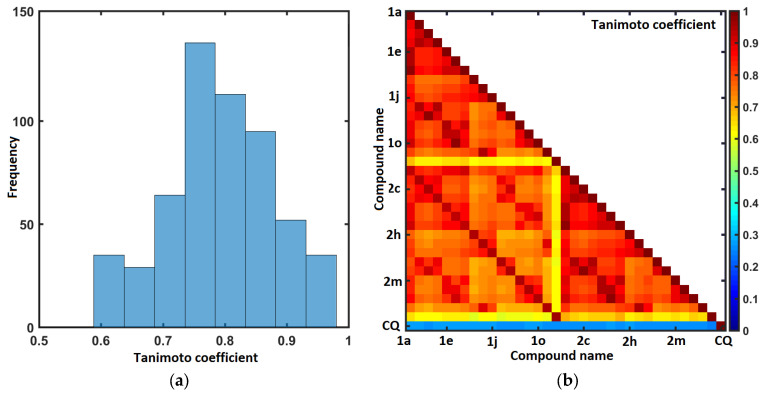
Distribution of Tanomoto coefficients (**a**) and triangular matrix of Tanimoto coefficients (**b**) for 4-chlorocinnamanilides **1a**-**q** and 3,4-dichlorocinnamanilides **2a**-**q** compared to chloroquine (CQ).

Conceptually, a numerical measure of molecular diversity between two objects can be quantitatively expressed by a bit-string representation (sometimes augmented with the scaling coefficients) in the function of (un-)common features. The pair-wise relatedness between descriptor-guided structures can be numerically evaluated by a variety of the ‘relative’ distance metrics (e.g., Hamming or Euclidean measures) and/or the ‘absolute’ comparison using Tanimoto coefficient calculated for molecular fingerprints (e.g., OpenBabel FPs) [55]. The distribution of Tanimoto coefficients was analyzed for the investigated series **1** and **2** with the highest frequency recorded in the relatively high similarity range of 0.78 < T < 0.88, as presented in Figure 4a. A triangular matrix of T_35×35_ shown in Figure 4b indicates the noticeable structural dissimilarities of bromo-substituted and trifluorometoxylated compounds **1q** and **2q** from the remaining ones. Not surprisingly, CQ varies structurally from the 4-chlorocinnamanilides **1a**–**q** and 3,4-dichlorocinnamanilides **2a**–**q**, that is marked by low values of Tanomoto coefficient.

**Figure 5:**
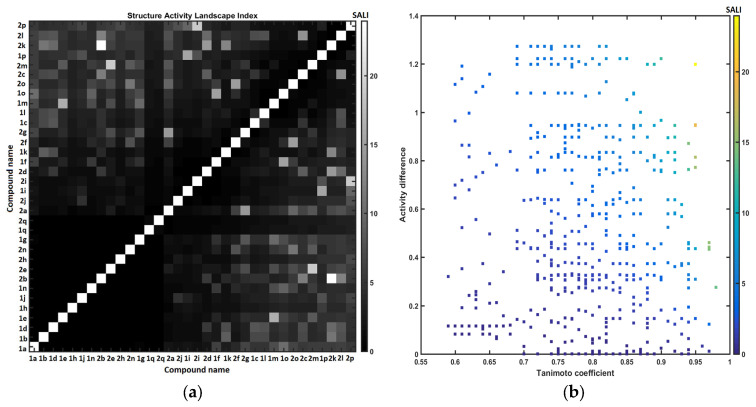
Gray-scaled SALI plot with molecules ordered by increasing antiplasmodial activity (**a**) and corresponding neighboring plot (**b**) for 4-chlorocinnamanilides **1a**-**q** and 3,4-dichlorocinnamanilides **2a**-**q**.

A systematic profiling of structure-activity landscape indexes (SALI) for the structurally-related molecules (chemotypes) with discernible activity variations enables a graphical representation of similarity-based SAR trends in the form of continuity areas and/or activity cliffs, respectively. Obviously, for similar molecules (e.g., stereoisomers where T→1) SALI→infinity; therefore, such values are replaced by the largest SALI value [56]. The symmetrical grayscaled heatmap of SALI values for the investigated series **1** and **2** of (di)chlorinated in C-phenyl ring cinnamanilides is presented in Figure 5a, where axes correspond to a compound name sorted according to the increasing antiplasmodial activities (ΔpP*_f_* ≈ 1.75) with a legend depicting the range of SALI values—white spots of the heatmap represent the highest numerical values of SALI parameters, while the black ones specify the minimal, respectively. In fact, the left side of the heatmap indicates the ‘smooth’ landscape regions with lower SALI values, which are generally occupied by the inactive molecules. Contrarily, the lighter blocks located in the right lower part of the heatmap (or symmetrically positioned in the upper left side) shows the pairs of molecules, that potentially can form the activity cliffs, where relatively huge variations in the inhibitory potency is manifested for the similar structures. Interestingly, for the most potent, di-substituted Cl/F/CF_3_-containing inhibitors **2**(**m**,**k**,**l**,**p**) and **1p**, the removal of one substituent from *N*-phenyl ring demolishes the antiplasmodial activity, that is marked by higher SALI parameter in Figure 5a (e.g., **2p**→**2i**, **2k**→**2b** or **1p**→**1i**). Moreover, the mentioned structural modifications, that unfavorably affect the affinity profile, can be tracked down on the neighborhood plot in Figure 5b, where the structurally related pairs of molecules are plotted versus differences in the biological activity and color coded by higher SALI values as well. The right upper side of the plot (T > 0.85 & ΔpP*f* > 1.0) indicates the ‘rough’ SALI regions, where the introduction of the additional fluorine substituent to the aromatic mono-substituted fluorine-based *N*-phenyl system resulted in the boost of the inhibitory potency (e.g., **1b**→**2k**, **1d**→**2k** or **2b**→**2k**), that is color coded by higher SALI values (see Figure 5b). Consequently, further profound samplings of the marked SAR-variations seem advisable to reveal the activity cliffs for the investigated arginase inhibitors.

#### 2.3.3. Probability-Guided Pharmacophore Mapping

**Figure 6:**
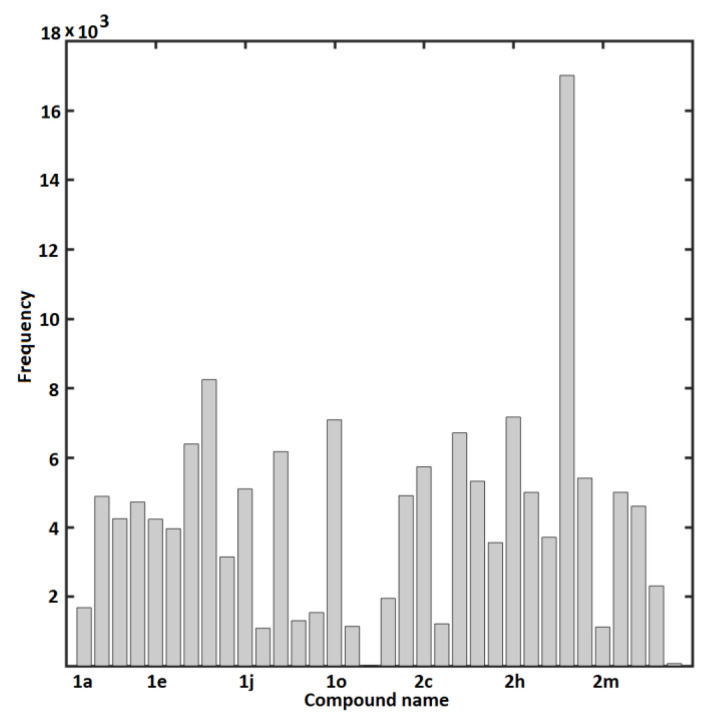
Frequency histogram of molecules **1a**-**q** and **2a**-**q** within test subset for valid SAR models.

In order to explore the spatial distributions of the ligand electronic and steric properties that might be valid for the inhibitor-arginase complex the systematic probing of the functional group changes and the related activity variations, we applied 3D-QSAR comparative molecular field analysis (CoMFA) and comparative molecular surface analysis (CoMSA), respectively [57]. It should be emphasized, that we did not concentrate on details of each modeling procedure (e.g., CoMFA or CoMSA), but more on the philosophy of molecular object descriptions (atom-based or surface-driven) [58]. In this case, both 3D methods perform comparably (CoMFA: $q_{cv}^{2}=0.73$ vs. CoMSA: $q_{cv}^{2}=0.76)$ for the whole set of the analyzed molecules. The exclusive reliance on the training subset (the internal validation with the cross–validation CV) is inadvisable to determine the robustness and the predictive ability of models [59]; therefore, the external model validation with splitting the molecule collection into the training/test subsets was performed with ≈ 4:1 ratio (28/6) using Kennard-Stone’s procedure. In both cases, the $q_{cv}^{2}$/$q_{test}^{2}$ outcome indicates a comparable efficiency in modeling of the drug inhibitory potency (CoMSA: 0.68/0.60 vs. CoMFA: 0.62/0.60 CoMFA). An additional question appears whether we can differentiate between modeling and the predictive model ability, knowing that the quality of models is considerably dependent on the classification of molecules into training/test subpopulations—no correlation between good retrospective performance and good prospective performance was observed in namely Kubinyi paradox [60]. In this context, restricting ourselves to single$\;q_{cv}^{2}$/$q_{test}^{2}$ numerical value can be misleading; therefore, we proposed the repetitive and interchangeable training/test subset division for the probability-driven pharmacophore probing called the stochastic model validation (SMV) algorithm [61]. Despite the CPU-intense SAR calculations, it was technically feasible to investigate the whole pool of systematically generated training/test populations ($C_{34}^{6}$*≈1.3×10^6^*) for CoMSA pP*_f_* inhibitory modeling. The frequency distribution of the test compounds in models with the preferable$\;q_{cv}^{2}\;$≥ 0.75 and $q_{test}^{2}$ ≥ 0.6 parameters revealed that the active molecule **2k** is noticeably over-represented, as illustrated in Figure 6. It means that the active molecules should be selected to the test population in order to map comprehensively the entire activity space. On the whole, the preferential selection of mono-/di-substituted active (P*_f_* IC_50_ < 5.0) and inactive molecules (P*_f_* IC_50_ > 5.0) is observed for the test subset that resulted in the generation of the robust models with the acceptable predictive power for the test set.

**Figure 7:**
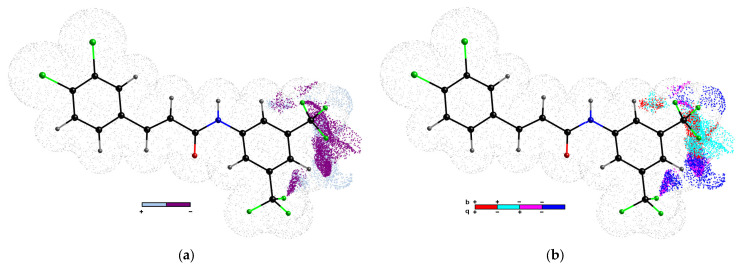
Spatial pattern identified by CoMSA IVE-PLS method for 28/6 training/test samplings of molecules **1a**-**q** and **2a**-**q** with antiplasmodial activity. Colors code the sign of contribution (**a**) and four possible combinations of mean charge and correlation coefficients are for charge descriptors (**b**). Compound **2p** was plotted as a reference molecule.

Next, the subsequent level of the variable reduction for the uninformative data (highly correlated descriptors) was employed to enhance the model interpretability using our IVE-PLS (iterative variable elimination partial least squares) method as a filter to eliminate non-significant variables (probably noise data) and to identify structural descriptors having the highest individual weightings for the biological activity. In consequence, an ‘averaged’ selection-driven interaction pattern was produced based on the regions of the pretty high model ability and predictability in namely ‘pseudo–consensus’ 3D pharmacophore mapping [62]. The graphical illustration of the descriptor-based areas that contribute (un)favorably into CoMSA models with the preselected cut-off value of 0.5 for molecular surface descriptors with an acceptable statistical importance is shown in Figure 7. The relative contribution of the surface/charge descriptors is weighted by the corresponding regression coefficient indicating the regions of the positive (bright color) and negative (dark color) impact on the inhibitory potency (see Figure 7a). Moreover, the four possible combinations of the charge (q) and the mean regression coefficients (b) are introduced in Figure 7b.

In general, the direct translation of the pharmacophore-related points in 3D space into the corresponding pseudoreceptor model with privileged zones, that potentially harbors putative inhibitors is fairly tricky task—an ‘averaged’ receptor structure is postulated using the ‘reverse image’ of the hypothetical pharmacophore electrostatic/steric pattern. Not surprisingly, the spatial distribution plot shown in Figure 7a demonstrates the (un)favorable steric contributions of areas that spread uniformly over *meta/para* substituted N-phenyl ring. Noticeably, the increase in the bulkiness at *para* position of N-phenyl ring appears to be unfavorable structural modification, as illustrated via the negative values of the dark areas next to the N-phenyl system of the most potent compound **2p**. It confirms the tendency recorded for mono-substituted isomers in series **1** and **2**, where inhibitory profile can be basically ranked according to the following relation *meta>>para>ortho* (see Table 1). Accordingly, the negatively charged spheres in Figure 7b with the corresponding positive regression coefficients mean, that some polar (electronegative) substituent/group (e.g., -F, -Cl or –CF_3_) at *meta* position of the N-phenyl ring contributes unfavorably to the antiplasmodial activity. Conversely, the relative significance of *meta* regions is marked by the gray 3D polyhedrals (see Figure 7a), where the negatively charged atoms were depicted as the favorable contributors to the inhibitory potency of the investigated molecules, as shown in Figure 7b. In other words, the increased electron density on halogen atoms or trifluoromethyl group in the close proximity of positions 3 and 5 in the *N*-phenyl group corresponds well with the increased antiplasmodial potential as recorded in Table 1. Oddly enough, the provided pharmacophoric pattern based on the consensus 3D-QSAR modeling does not explain the observed variations in the inhibitory potencies between monochlorinated arylcinnamamides (series **1a**–**q**) and the corresponding dichlorinated in C-phenyl ring analogues (series **2a**–**q**); therefore, the structure-based drug-design (SBDD) method was engaged as well.

#### 2.3.4. Molecular Docking Simulations

More detailed insight into the spatial guest-host interactions might be gained using the site-directed protein-based docking procedures, specifically useful, when an exact 3D receptor/enzyme geometry (or homology model) is available in the protein database. Regrettably, it is still not obvious how to correlate accurately the enthalpically and/or enthropically favorable ligand-receptor modes and scoring function values with the pharmacological or toxicological effects (ADMET profile) [63]. In practice, the utility of the intuitive docking procedures in the reconstruction of the guest-bound poses is commonly accepted as the comprehensive extension of the classical 3D ligand-based (LBDD) methods. As a matter of fact, the complementary ligand-receptor binding mode is deduced from the spatial arrangement of the target atoms using the feature/descriptor-matching algorithms, where the ligand property space is correspondingly mapped to the macromolecular steric, electrostatic and/or lipophilic features.

**Figure 8:**
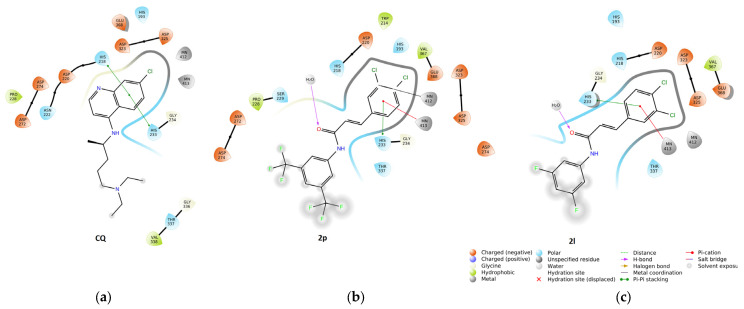
Planar ligand interaction diagram for chloroquine (**a**) and the most potent inhibitors **2p** (**b**) and **2l** (**c**) generated by Maestro program.

As far as we know, the exact mechanism of action of cinnamic acid scaffolds on *P. falciparum* remains unknown; therefore, we made in silico attempts to reconstruct the drug-protein interactions using the molecular docking simulations in order to obtain more potent cinnamic acid derivatives. Hence, the atomic coordinates of *P. falciparum* arginase (PFA) in complex with the boronic acid inhibitor (ABH) were downloaded from the European Protein Data Bank repository (PDBe code: 3mmr) [27]. The crystallographic geometry was determined by X-ray diffraction at a sophisticated resolution of 2.14 Å in the ligand-containing state (ABH-PFA holo structure); however, the whole structure was corrected and optimized in Sybyl-X Biopolymer package. Since the molecular recognition in guest-host system might be stimulated by water-mediated hydrogen bonds (HBs); therefore, six crystalic water molecules in the active site AC3 and AC4 have not been removed from PFA structure. Subsequently, the marketed drug CQ and the potential antiplasmodial agents (series **1** and **2**) were docked into the active sites AC1-AC4 of the arginase chain A using AutoDock Vina 1.2.0 software in order to collate the binding pattern of the (di)chlorinated *N*-arylcinnamamides with the CQ-PFA interacting mode [64,65]. It has been postulated previously that cinnamoyl or 3,4-hydroxy cinnamoyl motifs were crucial for the mechanism of arginase inhibition, where the catechol-like moiety was frequently involved in a metal (Mn^2+^) coordination bond by one of its hydroxyl groups [66]. Following this suggestion, it seems interesting how the introduction of one or two chlorine atoms in the aromatic ring of *N*-arylcinnamammides would affect the PFA-inhibitor interacting mode. Thus, the entire antiplasmodial population was docked and compared with the drug-enzyme (CQ-PFA) binding pattern using Schrödinger Maestro software and Protein-Ligand Interaction Profiler (PLIP) [67]. Despite some noticeable structural variations between CQ and the analyzed *N*-arylcinnamammides, some regularities in the spatial atomic distribution and non-bonding interactions (hydrogen bonds or hydrophobic pattern) can be observed, as shown for the most active molecules in Figure 8.

**Figure 9:**
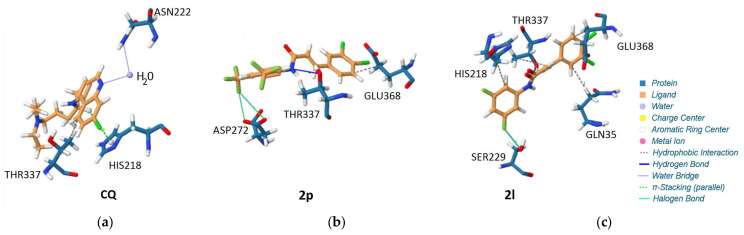
Spatial comparison of binding interactions for drug chloroquine (**a**) and the most potent inhibitors **2p** (**b**) and **2l** (**c**) generated by PLIP tool.

First of all, the chlorinated and dichlorinated C-phenyl rings are oriented towards the binuclear manganese cluster in the energetically favorable poses of CQ and the most potent arginase inhibitors **2p** and **2l**, as illustrated in the ligand interaction diagram (see Figure 8a–c). The analogous metal coordination tendency of the hydroxyl group(s) in the active site was reported for catechol-containing cinnamic and rosmarinic acid-derived antileishmanial agents [17,68]. Moreover, the face of the electron-rich aromatic component (C-phenyl ring) substituted with one or two chlorine atoms could constitute π-π stacking interactions with the histidine residues (His233 and/or His218) of PFA active site and the cation-π non-covalent interactions with the adjacent manganese cofactor (Mn^2+^). Additionally, the water-mediated hydrogen bonds were formed via carbonyl function present in the new *N*-arylcinnamamides, as shown for the most potent molecules **2p** and **2l** in Figure 8b,c. The intermolecular water bride could also bond the ligand atoms as well as the aminoacid residues, e.g., the aromatic nitrogen of CQ ring and the nitrogen of the asparagine (Asn222) side-chain (see Figure 9a). In fact, it confirmed the previously reported observations for hAI-ABH complex, where incorporation of water molecules into the arginase active site was valid for HB formation [30]. The spatial ligand interaction scheme presented in Figure 9b,c revealed the potency of the most active molecules **2p** and **2l** to form the hydrogen and halogen bonds as well.

The hydroxyl group of Thr337 residue was indicated to be potentially hydrogen-bonded (as hydrogen donor) with the amide nitrogen (as hydrogen acceptor) of the most active inhibitors **2p** and **2l**, respectively. Interestingly, the fluorine substituent (alone or in trifluoromethyl group) in *N*-phenyl ring seems to play a key role in forming the halogen bonds (see Figure 9b,c). Overall, the halogen bonds (or X-bonding) have become recognized recently as potential stabilizers of the inter-/intra-molecular interactions that contribute significantly to recognition specificity of the halogenated molecules (or halogen-containing metabolites), usually used as inhibitors against the biomedically important targets [69,70]. De facto, two electrostatically driven, highly directional and short halogen–oxygen interactions were specified between fluorine of –CF_3_ substituent in the most potent molecule **2p** and the proximal oxygen atoms of the carboxyl functional group in Asp272 residue. A similar X-bonding was recorded for the active inhibitor **2l**, where directly attached to N-phenyl ring fluorine atom interacts with one hydroxyl oxygen of Ser229 amino acid residue.

Although, there is no clear explanation of the antiplasmodial profile variances for the variously positioned *N*-arylcinnamamides provided by the docking study further exploration of the X-bonding pattern of the mono/di-chlorinated analogues seems reasonable.

## 3. Materials and Methods

### 3.1. Chemistry

All discussed (2*E*)-3-(4-chlorophenyl)-*N*-arylprop-2-enanilides **1a**–**1q** and (2*E*)-3-(3,4-dichlorophenyl)-*N*-arylprop-2-enanilides **2a**–**2q** were previously prepared and characterized by Strharsky et al. [26].

### 3.2. Lipophilicity Determination by RP-HPLC

Experimental determination of lipophilicity values (log*k*) of all discussed (2*E*)-3-(4-chlorophenyl)-*N*-arylprop-2-enanilides **1a**–**1q** and (2*E*)-3-(3,4-dichlorophenyl)- *N*-arylprop-2-enanilides **2a**–**2q** was described by Strharsky et al. [26]. The log*k* values of individual compounds are shown in Table 1.

### 3.3. In Vitro Cell Viability Analysis

Human monocytic leukemia cell line THP-1 obtained from the European Collection of Cell Cultures (ECACC, Salisbury, UK) was used for in vitro determination of the influence of test compounds on cell viability, as described previously [26]. The results are shown in Table 1.

### 3.4. In Vitro Antiplasmodial Activity

The following reagent was obtained through BEI Resources, NIAID, NIH: *Plasmodium falciparum*, strain 3D7, MRA-102, contributed by Daniel J. Carucci. Based on a modified procedure by Trager and Jensen [71], asexual erythrocytic stages of *Pf* were continuously maintained through in vitro culture. The chloroquine-sensitive strain is cultured thanks to human red blood cells (A+) and a culture medium mainly composed of RPMI 1640 (Gibco, Fisher Scientific, Loughborough, UK) containing NaHCO_3_ (32 mM), HEPES (25 mM), and l-glutamine. The medium was supplemented with 1.76 g/L of glucose (Sigma-Aldrich, Machelen, Belgium), 44 mg/mL of hypoxanthine (Sigma-Aldrich), 100 mg/L of gentamycin (Gibco, Fisher Scientific) and 10% human pooled serum (A+), as previously described [72]. Solutions of pure products were prepared in DMSO at 10 mg/mL. As DMSO is recognized as toxic for parasites, the highest concentration of solvent to which they were exposed was 1%. Thus, primary solutions were diluted in a culture medium to reach 100 µg/mL in the first row of a 96-well plate. Therefore, each test sample was applied in a series of eight 2-fold dilutions and tested in triplicate. The assay was performed with 2% parasitaemia and 1% haematocrit [73]. After 48 h of incubation, plates were frozen at −20 °C for 12 h and parasite growth was quantified according to the methods described by Makler et al. [74]. Chloroquine (Sigma-Aldrich) was used as positive standards in all experiments, with initial concentrations at 100 ng/mL. Infected and uninfected red blood cells (RBC) were used as positive (100% growth) and negative controls (0% growth). Consequently, comparison between infected erythrocytes and samples allowed us to estimate the growth inhibition. IC_50_ values were calculated from linear regression. Due to the great number of compounds to test, a first experiment was performed (twice) with one concentration = 50 µg/mL. The molecules that did not reach 45% of inhibition at 50 µg/mL were discarded. The results are shown in Table 1.

### 3.5. In Vitro Hemolytic Activity

Hemolysis induction was evaluated for all the tested compounds based on a reported procedure [75]. Consequently, a 10% red blood cell suspension in PBS (*v*/*v*) (A+) was incubated with compounds in duplicate. The primary solutions were diluted in PBS to reach 100 μg/mL as the final concentration (DMSO < 1%). After agitation at room temperature for 1 h, the mixtures were centrifuged for 5 min at 2000 rpm, and 150 μL of supernatant was transferred to a 96-microwell plate. The absorbance was evaluated at 550 nm with a microplate reader (OD). The positive control was Triton X-100 1% (*v*/*v*) (corresponding to 100% lysis), and PBS was the negative control (corresponding to 0% lysis). The percentage of red blood cell lysis (*H*) was calculated as follows: *H* = (*OD*_550_ sample − *OD*_550_ PBS)/(*OD*_550_ Triton X-100 1% (*v*/*v*) − *OD*_550_ PBS) × 100. The hemolysis was considered insignificant if it was lower than 1% of the total RBC.

### 3.6. Computational Details

#### 3.6.1. Ligand Building and Modeling

CACTVS/csed and CORINA editors were used to generate 3-dimensional molecular models of the ligand population. The data format conversion was conducted using OpenBabel (inter)change file format converter. Sybyl-X 2.0/Certara package installed on a DELL workstation with Ubuntu 20.10 operating system was employed to perform the molecular modeling simulations. Sybyl-X MAXMIN2 module was engaged to initially optimize the compound spatial geometry with the standard Tripos force field (POWELL conjugate gradient algorithm) with a 0.01 kcal/mol energy gradient convergence criterion. The electrostatic potential values were calculated using Gasteiger–Hückel method implemented in Sybyl-X package. One eleven-ordered atom trial alignment was applied on the most active compound **2p** (according to active analogue approach AAA) with FIT procedure to cover the entire bonding topology in the maximal common structure (MCS). SONNIA software was implemented to simulate self-organizing maps (SOMs) of size 10 × 10 to 30 × 30 with a winning distance in the range from 0.2 to 2.0. Molecular 3D geometry (x,y,z atomic coordinates) of the molecular surfaces and the corresponding potential values were used as an input to Kohonen SOM network in order to generate a 2D map of the electrostatic potential (MEP) in CoMSA analysis. The produced maps were reshaped into a 100- to 900-element vector subjected to the PLS method implemented in the MATLAB environment.

#### 3.6.2. Theoretical Lipophilicity Evaluation

A number of freely/commercially accessible in silico estimators might be engaged to calculate the theoretical partition coefficients (clogP), for instance:

AlogPS—approach proposed by Tetko et al. that is based on atom-type electrotopological-state (E-state) indices and neural networks (NN);

milogP—method implemented by Molinspiration for practical logP calculations of almost all organic molecules as a cumulative sum of the fragment-based contributions and the correction factors;

ClogP—fragment-based algorithm to predict the molecular lipophilic profile based on structure-dependent correction values derived from Hansch and Leo’s database that is implemented in Sybyl/Centara software;

HyperChem logP—an atom-additive methodology that approaches lipophilicity using the individual atomic contribution proposed originally by Ghose, Prichett and Crippen;

MarvinSketch logP—the overall lipophilic profile of a molecule is composed of the contributing values of its atom types that were redefined to accommodate electron delocalization and contributions of ionic forms;

ChemSketch logP—a comprehensive fragment-based algorithm with the high-quality models derived using empirical data. Well-characterized logP contributions were compiled for atoms, structural fragments and intramolecular interactions calculated for more than 12 × 10^3^ experimental logP values;

Dragon AlogP—the statistical predictor of Ghose–Crippen–Viswanadhan model, that was specified on the basis of known experimental logP for the training set of 8364 compounds. The overall estimation of the lipophilic atomic-based constant is evaluated with the contribution of 115 atom types;

Dragon MlogP—the theoretical partition coefficient includes VdW volume and Moriguchi polar parameters as correction factors. A regression MlogP model is based on 13 structural variables evaluated on the training group of 1230 organic molecules;

Kowwin—estimates the log octanol-water partition coefficient of chemicals using the atom/fragment contribution algorithm;

XlogP3—an atom-additive methodology with well-defined correction factors that used an optimized atom typing approach calibrated on a big training set;

OSIRIS clogP—in house approach based on the cumulative sum of atom contributions estimated for more than 5000 compounds with empirically determined logP values for the training set. Predicting engine distinguishes 369 atom types;

ChemBio clogP—the algorithms for estimation of partition coefficient based on a training set of compounds that provide coverage for a broad chemical space;

Percepta clogP—based on >12 × 10^3^ of experimental logP values with the algorithm that uses the principal of isolating carbons.

The redundant variables/descriptors in QSAR/QSPR investigations were selected/extracted by the modified version of the uninformative variable elimination (UVE–PLS) procedure, in namely iterative variable elimination (IVE–PLS) method. Briefly, the entire algorithm composes of the following stages: (i) standard PLS analysis with LOO–CV to evaluate the performance of the PLS model; (ii) elimination of the matrix column with the lowest *abs(mean(b)/std(b))* value; (iii) standard PLS analysis of the new matrix without the column eliminated in (ii); (iv) iterative repetition of (i)–(iii) to maximize $q_{cv}^{2}$ value.

#### 3.6.3. PCA, HCA and SALI Assessment

The human-friendly 2D/3D graphical presentation of the compound’s distribution in the experimental (FCS) and virtual (VCS) chemical space might be produced by the Principal Component Analysis (PCA). PCA is a linear projection methodology that can be employed to model multidimensional data (mDs) with a relatively small number of so-called principal components (PCs) produced to maximize the description of variance within the input data. The PCA model with *f* principal components (scores and loadings) for a data matrix *X* can be specified according to the following formula:

*X* = *TP^T^*+ *E* (1)

where *X* is a data matrix with *m* objects and *n* variables, *T* is the score matrix with dimensions (*m* × *f*), *P^T^* is a transposed matrix of loadings with dimensions (*f* × *n*) and *E* is a matrix of the residual variance (*m* × *n*) not explained by the first *f* principal components. In practice, the first few PCs frequently describe sufficient data variance and reveal the groups of similar objects.

Hierarchical Clustering Analysis (HCA) facilitates the examination of the (dis)similarities between objects in the multivariable chemical space. Hence, the similarity measure as well as the manner of sub-clusters linkage should be specified a priori. The generated outcome is displayed as a dendogram, where OX axis presents the indices of the clustered objects and OY axis corresponds to the linkage distances between two connected objects, respectively. Furthermore, the visualization method can be extended with the empirical data sorted according to the order of objects with the generation of the color-coded feature/property maps. A mutual interpretation of objects sorted with the Ward linkage method and the color-coded experimental data enables the (dis)similarity evaluation of objects/molecules in terms of the input parameters/descriptors.

The numerical profiling of the similarity-related structure-activity landscape index (SALI) can be quantitatively performed using the subsequent equation:

(2) $$SALI_{x,y}=\frac{\left|A_{x}-A_{y}\right|}{1-sim\left(x,y\right)}$$

where $A_{x}\;$and$\;A_{y}\;$are the activity profiles for the x-th and y-th molecule and *sim(x,y)* is the pair-wise similarity measure. Tanimoto coefficient was used for the fingerprint-based similarity evaluation, where the structural pair-wise molecular relatedness is calculated as follows:

(3) $$T\left(x,y\right)=\frac{n_{xy}}{\left(n_{x}+n_{y}-n_{xy}\right)}$$

where$\;n_{xy}$ is the number of bits set into 1 shared in the fingerprint of the molecule *x* and *y*, $n_{x}$ is the number of bits set into 1 in the molecule *x*, $n_{y}$ is the number of bits set into 1 in the molecule *y*, respectively.

#### 3.6.4. Ligand-Based Activity Modeling

Self-organizing maps (SOMs) conjugated with weighting and selecting procedures (IVE-PLS) were used to specify the minimal/optimal ensemble of pharmacophoric features that are potentially important in description of the guest–host interactions. The comparative molecular surface analysis (CoMSA) was engaged to directly compare/contrast the shape and charges distribution generated on the molecular surface of the ligands. Briefly speaking, a single layer of neurons arranged in a 2D plane with well-defined topology to produce self-organized maps (SOMs). The geometrically similar objects (analogues) are located in the proximal neurons of the square map in the process of SOMs adaptation to the input data. In consequence, 2D image of the property space is produced, where structurally related molecules are placed in neighboring neurons. The electrostatic/steric pattern that is potentially valid in the ligand-receptor complementarity and host recognition phenomena can be specified using the iterative variable selection approaches. Briefly, the backward column extraction is recurrently repeated until the optimal number of variables/descriptors included within the model is accomplished—the moment that the $q_{cv}^{2}$ deterioration specifies the ensemble of potentially relevant columns. The cumulative sum of the common columns/descriptors for the entire set of the investigated activity models was calculated and normalized to the range of [0–1]. Then, the columns that contribute (un)favorably into CoMSA models with the preselected cut-off value of 0.5 for molecular surface descriptors with an acceptable statistical importance are selected and graphically displayed on the atom/surface model of the most active molecule.

#### 3.6.5. Protein Preparation and Molecular Docking Simulations

The crystallographic structure of *Plasmodium falciparum* arginase in the liganded complex with co-crystalized ABH (2(*S*)-amino-6-boronohexanoic acid) and determined using X-ray diffraction at 2.14 Å resolution was downloaded from the PDBe repository (PDBe code: 3mmr). Apart from six crystalic water molecules in the active site AC3 and AC4 all remaining heteroatoms (including ABH molecule) were eradicated *prior* docking in AutoDock Vina 1.2.0 program. Initially, the ligand/enzyme structures were prepared in the pdbqt file format with the calculated Gasteiger charges. The grid box (size 15 × 15 × 15 Å) was centered on the central atom of ABH analogue. In AutoDock Vina, docking simulations different poses (default nine) were generated progressively from a single conformer (an energy-optimized molecule). The resulting molecular conformations and orientations with the preferred torsion angles and the rotatable bonds were then evaluated by the united-atom (UA) scoring function. Schrödinger Maestro graphical viewers and Protein-Ligand Interaction Profiler (PLIP) were employed to illustrate the foreseen 2D/3D binding modes, respectively.

## 4. Conclusions

In the presented study, we applied the collaborative (hybrid) protocol for the property mapping of novel chlorinated *N*-arylcinnamamides as potential PFA inhibitors. Initially, in vitro library screening of mono/di-chlorinated *N*-arylcinnamamides on a chloroquine-sensitive strain of *P. falciparum* 3D7/MRA-102 highlighted, that 23 compounds possessed IC_50_ < 30 µM and could be considered as active antiplasmodial agents. Subsequently, SAR-mediated similarity assessment of the structural descriptors and experimental data (inhibitory potential and lipophilic profile) for the new molecules was conducted.

High correlation between the experimental log*k* and estimated clogP was recorded for the whole ensemble of clogP generators. The backward *consensus* procedure indicated that HyperChem, Sybyl-X, ChemSketch, ChemBioDraw and Kowwin property predictors contribute significantly to the final lipophilic model. The projections of molecules **1a**–**q** and **2a**–**q** on the plane defined by PC1 vs. PC2 indicated, that the most active di-substituted with trifluoromethyl (3,5-CF_3_) moieties molecules **1p** and **2p** are located separately (PC1 > 75) from the rest of derivatives and are characterized by relatively high lipophilic values. The exploratory HCA approach produced the clustering pattern, where molecules are clustered into three main groups. Likewise, the most active 3,5-CF_3_ containing molecules **1p** and **2p** are grouped together in cluster A and are marked by higher values of lipophilicity. In order to predict the activity cliffs SALI indexes were calculated. Interestingly, for the most potent di-substituted Cl/F/CF_3_-containing inhibitors **2**(**m**,**k**,**l**,**p**) and **1p** the removal of one substituent from N-phenyl ring demolishes the antiplasmodial activity. On the other hand, the introduction of the additional fluorine substituent to the aromatic mono-substituted fluorine-based system resulted in the boost of the inhibitory potency. Moreover, the quantitative atom-based (CoMFA) and shape-related (CoMSA) ligand-oriented sampling of inter-molecular similarity and enzyme-driven molecular docking of ligands into the target pocket (active site) were applied to specify the electronic/steric/lipophilic factors and the ligand-enzyme (bio)composition that are potentially valid for the structure-inhibitory potency modeling of new PFA inhibitors. In consequence, an ‘averaged’ selection-driven interaction pattern was produced based on the regions of the pretty high model ability and predictability in namely ‘pseudo–consensus’ 3D pharmacophore mapping. Noticeably, the increase in the bulkiness at *para* position of N-phenyl ring appears to be unfavorable structural modification of the aromatic system for the most potent compound **2p**. It confirms the tendency recorded for mono-substituted isomers in series **1** and **2**, where inhibitory profile can be basically ranked according to the following relation: *meta>>para>ortho*. The increased electron density on halogen atoms or trifluoromethyl group in the close proximity of positions 3 and 5 in N-phenyl group corresponds well with the increased antiplasmodial potential.

The molecular docking approach was engaged for the most potent antiplasmodial agents in order to get comprehensive knowledge of the arginase-inhibitor binding mode. The docking study revealed that the (di)chlorinated C-phenyl rings are oriented towards the binuclear manganese cluster in the energetically favorable poses of the chloroquine (CQ) and the most potent arginase inhibitors **2p** and **2l**. Additionally, the water-mediated hydrogen bonds were formed via carbonyl function present in the new *N*-arylcinnamamides, that was shown for the most potent molecules **2p** and **2l**. Moreover, the spatial ligand interaction scheme indicated the potency of the most active molecules **2p** and **2l** to form the hydrogen and halogen bonds, respectively. The hydroxyl group of Thr337 residue was indicated to be potentially hydrogen bonded (as hydrogen donor) with the amide nitrogen (as hydrogen acceptor) of the most active inhibitors **2p** and **2l**, respectively. Interestingly, the fluorine substituent (alone or in trifluoromethyl group) of *N*-phenyl ring seems to play a key role in forming the halogen bonds. In fact, two short halogen–oxygen interactions were specified between fluorine of –CF_3_ substituent in the most potent molecule **2p** and the proximal oxygen atoms of the carboxyl functional group in Asp272 residue. A similar X-bonding was recorded for the active inhibitor **2l**, where directly attached to N-phenyl ring fluorine atom interacts electrostatically with one hydroxyl oxygen of Ser229 amino acid residue.

It seems that a collaborative combination of the pharmacophore mapping with target-tailored protocols can help to modulate pharmacological response and optimize ADMET-friendly drug properties to produce potentially more potent drug candidates.

## Figures and Tables

**Table 1:** Structures of ring-substituted (2*E*)-3-(4-chlorophenyl)-*N*-arylprop-2-enanilides **1a**-**1q**, (2*E*)-3-(3,4-dichlorophenyl)-*N*-arylprop-2-enanilides **2a**-**2q**, experimentally determined values of lipophilicity log*k*, in vitro antiplasmodial activity (IC_50_ [µM] ± SD, *n* = 3) against *P. falciparum* 3D7/MRA-102 (*Pf*) compared to chloroquine (CQ), and in vitro cell viability (IC_50_ [µM] ± SD, *n* = 3) on human monocytic leukemia cell line THP-1.

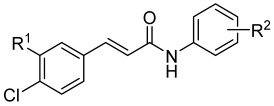					
No.	R^1^	R^2^	log*k* [26]	THP-1 IC_50_ [µM] [26]	*Pf* IC_50_ [µM] ± SD
**1a**	H	H	0.4490	>10	>30
**1b**	H	2-F	0.6459	>10	>30
**1c**	H	3-F	0.7859	>10	6.9 ± 2.1
**1d**	H	4-F	0.7808	>10	>30
**1e**	H	2-Cl	0.5241	>10	>30
**1f**	H	3-Cl	0.6192	>10	12.7 ± 2.1
**1g**	H	4-Cl	0.5598	>10	29.2 ± 2.4
**1h**	H	2-CF_3_	0.5506	>10	>30
**1i**	H	3-CF_3_	0.8612	>10	14.8 ± 3.1
**1j**	H	4-CF_3_	0.9078	>10	>30
**1k**	H	2,4-F	0.5746	>10	11.0 ± 2.1
**1l**	H	3,5-F	0.8054	>10	5.2 ± 0.9
**1m**	H	2,4-Cl	0.9665	>10	4.6 ± 1.8
**1n**	H	2,5-Cl	0.9507	>10	>30
**1o**	H	3,5-Cl	1.1899	>10	4.4 ± 1.8
**1p**	H	3,5-CF_3_	1.3064	>10	2.5± 0.9
**1q**	H	2-Br-4-OCF_3_	1.0278	>10	24.8 ± 2.5
**2a**	Cl	H	0.6199	>10	21.4 ± 2.6
**2b**	Cl	2-F	0.7764	>10	>30
**2c**	Cl	3-F	0.9071	>10	3.4 ± 1.0
**2d**	Cl	4-F	0.9009	>10	14.1 ± 3.1
**2e**	Cl	2-Cl	0.6488	>10	>30
**2f**	Cl	3-Cl	0.7404	>10	10.5 ± 2.0
**2g**	Cl	4-Cl	0.6786	>10	7.9 ± 1.7
**2h**	Cl	2-CF_3_	0.6836	>10	>30
**2i**	Cl	3-CF_3_	0.9873	>10	14.2 ± 2.3
**2j**	Cl	4-CF_3_	1.0364	>10	16.0 ± 2.5
**2k**	Cl	2,4-F	0.6989	>10	1.9 ± 0.06
**2l**	Cl	3,5-F	0.9298	>10	1.8 ± 0.08
**2m**	Cl	2,4-Cl	1.0932	>10	3.4 ± 0.9
**2n**	Cl	2,5-Cl	1.0840	>10	>30
**2o**	Cl	3,5-Cl	1.3043	>10	3.8 ± 1.1
**2p**	Cl	3,5-CF_3_	1.4731	>10	1.6 ± 0.07
**2q**	Cl	2-Br-4-OCF_3_	1.2065	>10	23.0 ± 2.7
**CQ**	–	–	–	–	0.5 ± 0.2

## Data Availability

The data presented in this study are available upon request from the corresponding authors.
